# Personalised approach to venous thromboembolism prophylaxis in heavier critically ill patients (PROSPER): A randomised, parallel-group, electronic medical record (EMR)-embedded feasibility trial

**DOI:** 10.1016/j.ccrj.2026.100229

**Published:** 2026-10-27

**Authors:** Yasmine Ali Abdelhamid, Fatima Ali, Amalia Karahalios, Haustine Patt Panganiban, Nadine K. Phillips, Timothy N. Fazio, Melissa J. Ankravs, Lucy Sharrock, Brianna Tascone, James R. Anstey, Christopher M. MacIsaac, Jeffrey J. Presneill, Adam M. Deane

**Affiliations:** a University of Melbourne, Melbourne Medical School, Department of Critical Care, Melbourne, Australia; b Royal Melbourne Hospital, Intensive Care Unit, Melbourne, Australia; c University of Melbourne, Melbourne School of Population and Global Health, Centre of Epidemiology and Biostatistics, Melbourne, Australia; d Methods and Implementation Support for Clinical Health Research (MISCH) Hub, Faculty of Medicine, Dentistry and Health Sciences, University of Melbourne, Melbourne, Australia; e University of Melbourne, Melbourne Medical School, Department of Medicine, Royal Melbourne Hospital, Melbourne, Australia; f Royal Melbourne Hospital, Clinical Informatics Centre, Melbourne, Australia; g Royal Melbourne Hospital, Pharmacy Department, Melbourne, Australia

**Keywords:** Electronic health record, Enoxaparin, Obesity, Overweight, Venous thromboembolism

## Abstract

**Objective:**

Data are limited regarding optimal low molecular weight heparin dosing for venous thromboembolism prophylaxis in heavier critically ill patients. This trial evaluated the feasibility of a weight-based dynamic dosing algorithm.

**Design, setting, participants:**

Open-label, electronic medical record-embedded randomised feasibility trial. Patients, weighing ≥100 kg who required thromboprophylaxis were eligible. Enoxaparin was commenced with weight-based dosing and anti-Xa level-guided adjustment (target peak anti-Xa level 0.2–0.4 IU/mL) or usual care (40 mg twice daily).

**Main outcome measures:**

The primary outcome was peak anti-Xa level at ‘steady state’ (after ≥3 enoxaparin doses). The feasibility threshold was set at 95% of steady state anti-Xa levels in the intervention group being less than the threshold for therapeutic anticoagulant effect (≤0.6 IU/mL).

**Results:**

Fifty participants (intervention n = 17, control n = 33) received 331 doses of enoxaparin (median of 4 [interquartile range {IQR}: 3–7] doses per participant, and a dose of 50 [IQR: 50–60] mg (intervention) and 40 [IQR: 40-40] mg (control)). Thirty-two ‘steady state’ peak anti-Xa measurements were recorded from 24 patients (12 intervention and 12 control). The proportion of peak anti-Xa levels that were ≤0.6 IU/mL was: 15 of 17 (88%) vs. 15 of 15 (100%). There were fewer peak anti-Xa measurements below the prophylactic range (<0.2 IU/mL) in the intervention group compared with the control group (2 of 17 [12%] vs. 5 of 15 [33%]). Six patients developed venous thrombosis or pulmonary embolism (2 of 17 [12%] vs. 4 of 33 [12%]). Major bleeding occurred in one patient in the intervention group.

**Conclusions:**

A weight-based dynamic thromboprophylaxis algorithm was implemented via the electronic medical record but did not meet the predefined feasibility threshold.

## 1 Background

During critical illness venous thromboembolism occurs frequently, is potentially preventable, and is associated with substantial morbidity and mortality.[1], [2], [3], [4], [5], [6]

In Australia, adults living with being overweight or living with obesity, represent approximately two-thirds of the adult population, and the mean body mass index (BMI) of critically ill patients admitted to the intensive care unit (ICU) has almost reached the obese category.^7^ Patients with overweight or obesity are at greater risk of venous thromboembolism.[8], [9], [10], [11], [12]

Prophylactic subcutaneous administration of a low molecular weight heparin mitigates the risk of venous thromboembolism in critical illness.^13^^,^^14^ Even in health, excessive weight is known to affect the pharmacokinetics and, thereby, the efficacy of low molecular weight heparin.^15^^,^^16^ Moreover, the pharmacokinetics of subcutaneously administered drugs are less predictable during critical illness, increasing the risk of sub-therapeutic drug concentrations.[17], [18], [19], [20]

Trial data evaluating the use of low molecular weight heparin for venous thromboembolism prophylaxis in critically ill patients who weigh ≥100 kg are limited.^10^^,^^21^ A post-hoc analysis of a dataset generated by a large international randomised clinical trial identified that increasing BMI was associated with failure of venous thromboembolism with prophylactic heparin.^11^ This has led to uncertainty about optimal dosing regimens in this group.[22], [23], [24] Furthermore, while inadequate dosing will increase the risk of thrombosis, excessive dosing will increase the risk of bleeding, and both conditions have significant consequences for patients.^25^

Given these factors, we conducted a clinical trial to determine the feasibility of a weight-based dynamic algorithm for venous thromboembolism prophylaxis in critically ill patients who weighed ≥100 kg. We hypothesised that a novel weight-based approach, with dynamic adjustment of enoxaparin dosing based on peak anti-factor Xa (anti-Xa) levels, would result in 95% of peak anti-Xa levels being less than the threshold for therapeutic anticoagulant effect (0.6 IU/mL).

## 2 Methods

### 2.1 Trial design

We conducted an investigator-initiated, single-centre, parallel-group, open-label, electronic medical records (EMR)-embedded, randomised clinical trial. The protocol was approved by the Human Research Ethics Committee of the Royal Melbourne Hospital, Australia (HREC/78209/MH-2021 2021.261). The trial was completed with a waiver of consent. The trial was prospectively registered on 02 February 2022 with the Australian and New Zealand Clinical Trials Registry (ACTRN12622000182796). We used the CONSORT statement extension for the reporting of feasibility trials.^26^

The trial was supported by an unrestricted grant from the Clive and Vera Ramaciotti Foundation. The funder had no role in the design or conduct of the trial, collection of the data, or analysis, interpretation, and presentation of the results.

### 2.2 Trial site and patient population

The trial was conducted at the Royal Melbourne Hospital. Patients admitted to the ICU aged 18 years of age or older, with a recorded weight of ≥100 kg, and who were deemed suitable to receive pharmacological venous thromboembolism prophylaxis with enoxaparin by the treating physician, were identified via the EMR. The exclusion criteria included: renal impairment (estimated glomerular filtration rate [eGFR] of <30 mL/min); admission after neuro-, cardiac- or spinal surgery; pregnancy; abnormal baseline coagulation (international normalised ratio [INR] >1.5 or activated partial thromboplastin time (APTT) >60 s or platelets <50 × 109/L); need for therapeutic anticoagulation; the treating doctor believed either a fixed-dose or a weight-based dynamic dose-adjustment regimen was required; or the patient had already received >3 doses of enoxaparin in the ICU.

### 2.3 Screening and randomisation

We used Epic (Epic Systems Corporation, Verona, Wisconsin, United States) and embedded screening, randomisation and adherence to treatment allocation as previously described.^27^ In brief, we utilised a randomisation schedule generated within the EMR. The EMR screened each patient against all inclusion criteria when the doctor started writing a prescription for enoxaparin. The EMR randomised patients who met all inclusion criteria in a 1:1 ratio to the intervention or control groups using a real-time number generator. The doctor was then prompted by the EMR to check all exclusion criteria. If no exclusion criterion was met, the physician could authorise the enoxaparin prescription and the patient was then enrolled in the study.^28^ Allocation concealment was maintained until the treating physician assessed the exclusion criteria, elected to include the patient in the study and was ready to authorise the enoxaparin prescription. Within Epic, only simple randomisation was available, with no capacity for stratification or block randomisation. Trial staff checked each enrolled patient daily to ensure that no enrolled patient met any exclusion criteria. Erroneously enrolled patients who met one or more exclusion criteria were removed from the trial and did not contribute to the intention to treat cohort.

### 2.4 Intervention and comparator

Both groups received subcutaneous enoxaparin as prophylaxis for venous thromboembolism. Those assigned to the intervention received enoxaparin according to a personalised algorithm. The personalised algorithm commenced with a weight-based dose of approximately 0.5 mg/kg twice daily (i.e., 50 mg twice daily for 100–119 kg to a maximum of 90 mg twice daily for a weight ≥180 kg) (Fig. 1). The personalised algorithm then used dynamic dose adjustment based on results of a peak anti-Xa level that was taken 4–6 h after the third dose (Fig. 1).

**Fig. 1:**
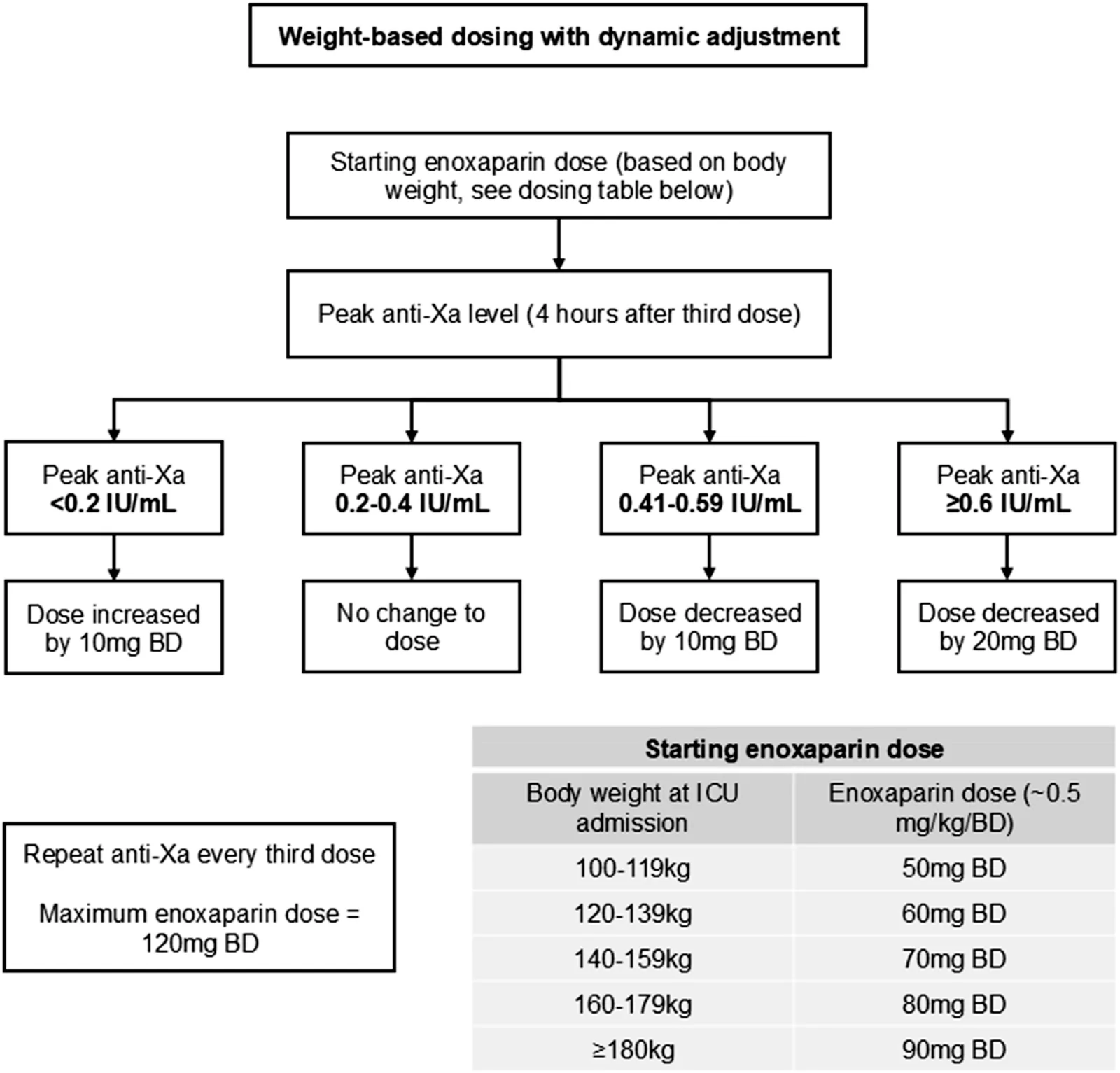
Intervention of personalised algorithm for enoxaparin that commenced with an initial weight-based dose with dynamic adjustment of subsequent dosing based on anti-Xa level. BD = twice daily.

Those assigned to the comparator received enoxaparin 40 mg twice daily, which was the unit protocol for patients weighing ≥100 kg and differed from dosing for patients weighing <100 kg (40 mg once daily). No dose adjustment was made for those in the comparator group.

Study participants remained in their allocated group for a maximum of 28 days or until they were discharged from the ICU, died, the treating intensivist believed a particular venous thromboembolism prophylaxis regimen was indicated at any stage (this was recorded as a protocol violation), the eGFR was <30 mL/min, there was a need for therapeutic anticoagulation, or the participant no longer had an arterial or venous catheter in-situ, such that blood for anti-Xa levels would require venepuncture.

### 2.5 Outcomes

Peak anti-Xa levels were measured for each participant at four to 6 h after the first and every third dose of enoxaparin administered.^29^ The primary outcome was the peak anti-Xa levels at ‘steady state’ (defined as after three or more doses of enoxaparin) with feasibility set at 95% of peak steady state anti-Xa levels being less than the threshold for therapeutic anticoagulant effect (0.6 IU/mL).^29^

Secondary outcome measures included:

• Peak anti-Xa levels prior to steady state (after the first or second dose of enoxaparin).
• The number of protocol violations (number of doses of enoxaparin administered that were incorrect according to the study protocol).
• Biochemical data; highest APTT and INR and lowest platelet and haemoglobin concentrations in the ICU and censored at day 14.
• Thrombosis: deep venous thrombosis and pulmonary embolism that were diagnosed in hospital (censored at day 90).
• Bleeding complications: that occurred in the ICU and were categorised as minor if venous thromboembolism prophylaxis was held or reduced, and major if accompanied by transfusion or an operation.
• Process of care: Blood transfusion and use of mechanical calf compressors.
• Clinical outcomes: duration of admission (ICU and hospital) and hospital mortality.

### 2.6 Statistical analyses

Data are presented as median (interquartile range [IQR]) for continuous measures and number (percentage) for categorical measures. For the primary outcome measure, the number (%) of peak anti-Xa levels at steady state that were in the ‘therapeutic systemic anticoagulation’ range (>0.6 IU/mL)^30^ and ‘prophylactic anti-Xa level range’ (0.2–0.4 IU/mL)^31^ were calculated. It was assumed that clinicians would be concerned about the intervention if the peak anti-Xa level was in the ‘therapeutic systemic anticoagulation’ range (>0.6 IU/mL).^30^ For this reason, the feasibility threshold was set at 5% of all anti-Xa levels obtained being in the therapeutic range at steady state in the intervention group, i.e., if the percentage of anti-Xa levels ≤0.6 IU/mL was 95% or more of recorded values, then the intervention of using a weight-based dosing regimen with dynamic adjustment was deemed feasible.

As a secondary outcome, because not all patients stayed in the ICU long enough to reach ‘steady state’, the number (%) of all anti-Xa levels that were in the specified therapeutic and prophylactic ranges were also calculated, including anti-Xa levels after the first or second dose of enoxaparin for each patient.

For the biochemical data, the highest APTT and INR values and the lowest platelet and haemoglobin concentrations by treatment group were summarised. For bleeding complications, processes of care and mortality, the number (%) with and without the events (i.e., minor and major bleeding complications, blood transfusion, use of mechanical calf compressions, and mortality) are presented by treatment group. For length of stay, the duration of ICU and hospital length of stay are summarised using median (25th, 75th percentiles). Modified intention-to-treat analyses were performed, excluding patients who met exclusion criteria but were erroneously randomised.

Subgroup analyses for the primary outcome were prespecified for two groups: patients who received at least 4 doses of enoxaparin in the ICU and patients who weighed ≥150 kg.

Given the lack of consensus as to how to determine sample sizes for feasibility studies,^32^ and the uncertainty regarding how many patients would meet all eligibility criteria and be randomised within the EMR, and the duration of exposure to drug prophylaxis in the ICU of this cohort, we chose to conduct this feasibility study over a 12-month period.

## 3 Results

### 3.1 Study participants

From 16 March 2022 to 15 March 2023, the EMR notified clinicians of 355 patients who met the inclusion criteria. When prompted by the EMR, clinicians identified that 302 patients met at least one exclusion criterion, with prescriptions signed and enrolment occurring for 53 patients (Fig. 2). Three patients had an estimated glomerular filtration rate of less than 30 mL/min at baseline, and so had been incorrectly randomised. These patients were withdrawn from the study by trial staff on the first study day, their Epic trial order set was discontinued, and they did not have anti-Xa levels measured. The study cohort of 50 included 17 participants in the intervention and 33 participants in the comparator group (Fig. 2). The intervention group was slightly younger than the control group with no other marked between-group differences at baseline (Table 1). There were no cases where a participant remained in ICU and no longer had an arterial or venous catheter in-situ and had to cease study medication.

**Fig. 2:**
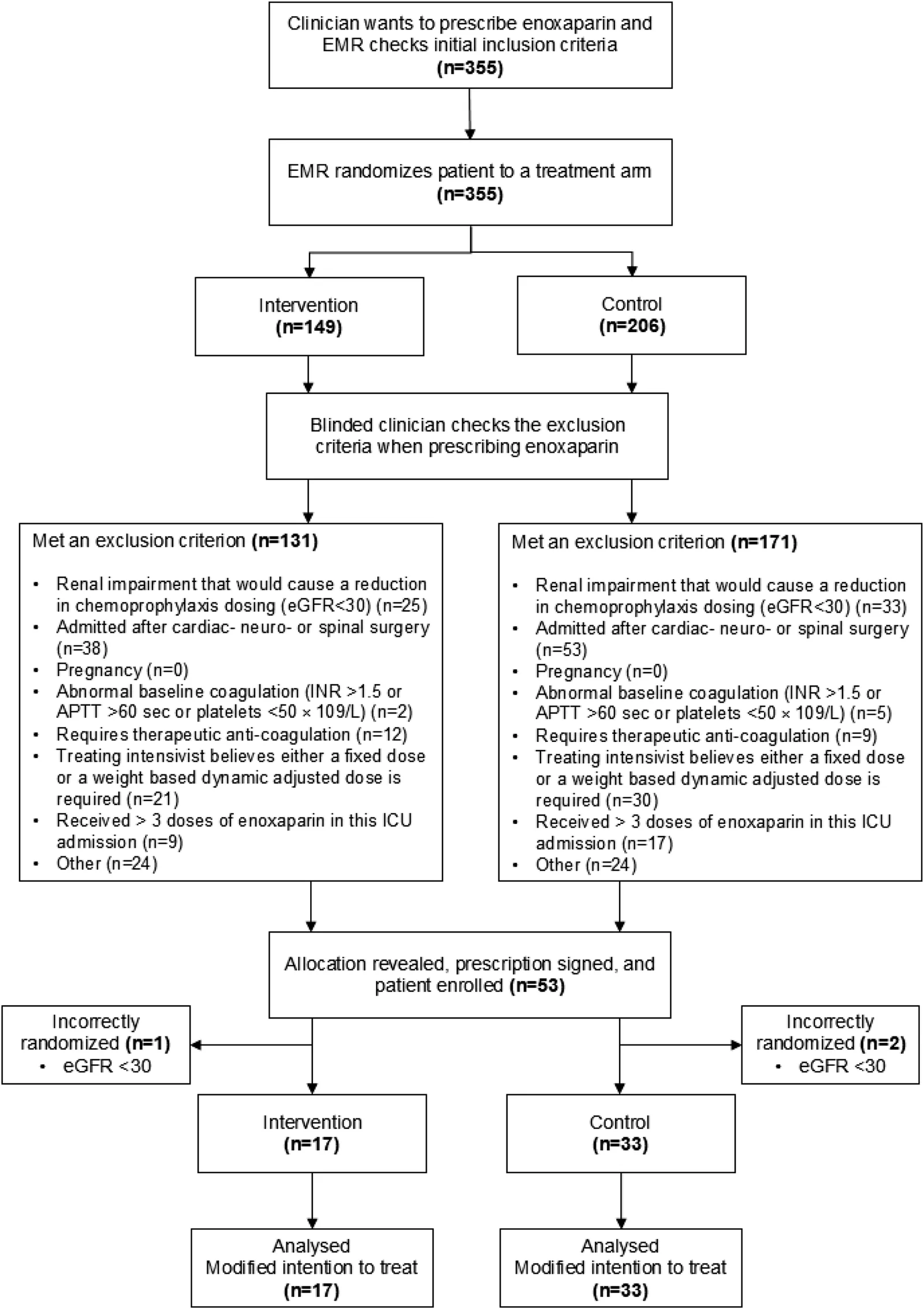
Patient flow diagram. Three patients met the exclusion criterion for renal impairment but were incorrectly enrolled and randomised by clinicians. These patients were withdrawn from the trial by trial staff for patient safety. EMR = Electronic Medical Record, INR = International Normalised Ratio, APTT = Activated Partial Thromboplastin Time, eGFR = estimated Glomerular Filtration Rate.

**Table 1:** Baseline characteristics of trial participants.

Baseline characteristic	Intervention (n = 17)	Control (n = 33)	Total (n = 50)
Age (years)	44.8 [35.0–53.4]	49.0 [36.6–59.0]	46.8 [35.0–58.0]
Sex			
Female	5 (29%)	12 (36%)	17 (34%)
Male	12 (71%)	21 (64%)	33 (66%)
Body weight (kg)	115.0 [108.0–127.8]	122.9 [106.0–132.0]	119.0 [106.0–132.0]
APACHE III score	39.0 [33.0–66.0]	40.0 [33.0–50.0]	40.0 [33.0–60.0]
ANZROD 2020 score	0.02 [0.01–0.09]	0.02 [0.01–0.07]	0.02 [0.01–0.07]
APACHE IIIj diagnostic category			
Nonoperative (medical)	8 (47%)	17 (52%)	25 (50%)
Nonoperative (trauma)	3 (18%)	6 (18%)	9 (18%)
Postoperative (surgical)	6 (35%)	5 (15%)	11 (22%)
Postoperative (trauma)	0 (0%)	5 (15%)	5 (10%)
Comorbidities			
Cancer	2 (12%)	1 (3%)	3 (6%)
Diabetes	4 (24%)	6 (18%)	10 (20%)
Frailty score			
Managing well	9 (53%)	17 (52%)	26 (52%)
Mildly frail	3 (18%)	9 (27%)	12 (24%)
Moderately frail	0 (0%)	2 (6%)	2 (4%)
Very fit	1 (6%)	0 (0%)	1 (2%)
Vulnerable	3 (18%)	3 (9%)	6 (12%)
Well	1 (6%)	2 (6%)	3 (6%)
Serum creatinine (μmol/L)^a^	73.0 [61.0–97.0]	81.5 [68.5–109.0]	80.0 [68.0–107.0]
Haemoglobin (g/L)^b^	131 [121–148]	128 [116–142]	128 [119–145]
APTT (seconds)^c^	31.0 [27.5–33.5]	29.0 [27.0–33.0]	30.0 [27.0–33.0]
INR^d^	1.0 [1.0–1.1]	1.0 [1.0–1.1]	1.0 [1.0–1.1]
Platelet count (x 10^9^/L)^e^	243 [184–316]	274 [216–310]	258 [214–312]
ICU source of admission			
Emergency department	8 (47%)	13 (39%)	21 (42%)
ICU other hospital	0 (0%)	1 (3%)	1 (2%)
Operating theatre recovery	5 (29%)	10 (30%)	15 (30%)
Other hospital	2 (12%)	2 (6%)	4 (8%)
Ward	2 (12%)	7 (21%)	9 (18%)

Data are presented as median [25th – 75th percentile] for continuous measures and number (%) for categorical measures.

APACHE = Acute Physiology and Chronic Health Evaluation, ANZROD = Australian and New Zealand Risk of Death, APTT = activated partial thromboplastin time, INR = international normalised ratio, ICU = intensive care unit.

a Serum creatinine has 1 missing value (1 control).

b Haemoglobin has 1 missing value (1 control).

c APTT has 7 missing values (6 control, 1 intervention).

d INR has 4 missing values (4 control).

e Platelet count has 1 missing value (1 control).

### 3.2 Processes of care

Processes of care are described in Table 2. The 50 participants received a total of 331 doses of enoxaparin (median of 4 [3–7] doses of enoxaparin per patient). The median dose of enoxaparin administered was intervention was 50 (IQR: 50–60) mg and comparator was 40 (IQR: 40-40) mg. Of the 13 participants who did not receive at least 3 doses of enoxaparin, 10 were discharged from the ICU before the third dose and 3 had deterioration in kidney function (GFR <30 mL/min).

**Table 2:** ICU processes of care.

Process of care	Intervention (n = 17)	Control (n = 33)	Total (n = 50)
Mechanical ventilation	11 (65%)	23 (70%)	34 (68%)
Duration of mechanical ventilation (hours)	61.1 [10.4–86.5]	75.6 [23.9–123.3]	70.1 [17.4–113.0]
Mechanical calf compression	2 (12%)	10 (30%)	12 (24%)
Total doses of enoxaparin received in ICU per patient	4 [3–6]	5 [3–8]	4 [3–7]
Dose of enoxaparin administered (mg)	50 [50–60]	40 [40-40]	–
Patients with peak anti-Xa level recorded at steady state	12 (71%)	12 (36%)	24 (48%)
Patients with peak anti-Xa level recorded at any time during study	13 (76%)	27 (81%)	40 (80%)
Number of anti-Xa levels per patient	1 [1–2]	1 [1-1]	1 [1-1]

Data are presented as median [25th – 75th percentile] for continuous measures and number (%) for categorical measures.

ICU = intensive care unit.

Ten participants did not have a correctly timed peak anti-Xa measurement at any stage, but the rest had at least one measurement (one, n = 29; two, n = 8; and three, n = 3). Missing measurements were due to clinical staff not taking blood samples, clinical staff taking blood samples at the wrong time in relation to the enoxaparin dose, or ICU discharge before an anti-Xa level was due.

### 3.3 Primary outcome

There was a total of 32 peak anti-Xa measurements recorded from 24 participants at ‘steady state’ (after 3 or more doses of enoxaparin) that contributed to the primary feasibility outcome (intervention: 17 measurements from 12 participants, comparator: 15 measurements from 12 participants). There were no substantive differences in baseline variables between these 24 participants and the other 26 participants who did not contribute to the primary feasibility outcome (Supplemental Table 1).

The proportion of peak anti-Xa levels at ‘steady state’ that were less than or equal to 0.6 IU/mL was 15 of 17 (88%) in the intervention group and 15 of 15 (100%) in the control group. This did not meet the predefined feasibility criteria for the primary outcome.

The proportion of peak anti-Xa measurements at ‘steady state’ in the ‘prophylactic anti-Xa level range’ (0.2–0.4 IU/mL) was 11 of 17 (65%) in the intervention group and 9 of 15 (60%) in the control group. There were fewer peak anti-Xa measurements at ‘steady state’ below the prophylactic range (<0.2 IU/mL) in the intervention group when compared with the control group (2 of 17 [12%] vs. 5 of 15 [33%]).

### 3.4 Secondary outcomes

#### 3.4.1 Other feasibility outcomes

There was a total of 54 peak anti-Xa measurements recorded for 40 participants, including peak anti-Xa measurements that were taken prior to ‘steady state’ (19 measurements from 13 participants in the intervention group and 35 measurements from 27 participants in the control group). The proportions of all recorded peak anti-Xa measurements below the prophylactic range were 3 of 19 (16%) in the intervention group and 17 of 35 (49%) in the control group. Additional ranges are presented in Supplemental Table 2.

Protocol deviations are described in Supplemental Table 3. The number of doses that were correctly administered according to the study protocol was 65 of 73 (89%) in the intervention group and 258 of 258 (100%) in the control group.

#### 3.4.2 Thrombosis, bleeding, and transfusion outcomes

Thrombosis, bleeding and transfusion outcomes are presented in Table 3. The proportion of patients who had deep venous thrombosis or pulmonary embolism diagnosed was 2 of 17 (12%) in the intervention group and 4 of 33 (12%) in the control group.

**Table 3:** Clinical and biochemical outcomes.

Outcome	Intervention (n = 17)	Control (n = 33)	Total (n = 50)
**Clinical outcomes**			
PE or DVT	2 (12%)	4 (12%)	6 (12%)
Major bleeding^a^	1 (6%)	0 (0%)	1 (2%)
Minor bleeding^a^	0 (0%)	0 (0%)	0 (0%)
Received blood transfusion	1 (6%)^b^	1 (3%)^c^	2 (4%)
Blood transfused (units)	8 [8-8]	1 [1-1]	4.5 [1–8]
Duration of ICU admission (days)	2.8 [1.7–3.7]	3.5 [1.7–4.8]	2.8 [1.7–4.6]
Duration of hospital admission (days)	6.5 [4.5–9.8]	7.7 [4.6–14.4]	7.6 [4.6–13.7]
Survived to ICU discharge	15 (88%)	31 (94%)	46 (92%)
Survived to hospital discharge	15 (88%)	30 (91%)	45 (90%)
**Biochemical outcomes**			
Highest APTT in ICU (seconds)	33.0 [31.0–37.0]	33.0 [30.5–35.0]	33.0 [31.0–36.0]
Highest INR in ICU	1.10 [1.00–1.10]	1.10 [1.10–1.15]	1.10 [1.00–1.10]
Lowest platelet count in ICU (x 10^9^/L)	212 [161–257]	199 [179–267]	206 [175–267]
Lowest haemoglobin in ICU (g/L)	111 [106–128]	105 [92–123]	108 [96–124]

Data are presented as median [25th – 75th percentile] for continuous measures and number (percentage) for categorical measures.

PE = pulmonary embolism, DVT = deep venous thrombosis, ICU = intensive care unit, APTT = activated partial thromboplastin time, INR = international normalised ratio.

a Bleeding defined as minor if venous thromboembolism prophylaxis was held or reduced, and major if accompanied by transfusion or operation.

b Associated with bleeding episode.

c Not associated with bleeding episode.

One major bleeding episode occurred in the intervention group (6%) and none in the control group. There were no minor bleeding episodes.

#### 3.4.3 Biochemical outcomes

The highest APTT and INR and lowest haemoglobin and platelet count in the ICU were similar between groups (Table 3).

#### 3.4.4 Other clinical outcomes

The durations of ICU and hospital admission and proportions of patients surviving to hospital discharge are provided in Table 3.

#### 3.4.5 Subgroup analyses

For the prespecified subgroup analysis of the cohort of patients who received at least 4 doses of enoxaparin in the ICU during the study, the anti-Xa profiles at steady state are provided in Supplemental Table 4. The results were similar to the primary analysis. Because only three patients weighed ≥150 kg no analysis was performed in this subgroup.

## 4 Discussion

In this trial, a weight-based dynamic algorithm for pharmacological venous thromboembolism prophylaxis in heavier critically ill patients weighing ≥100 kg was implemented in the ICU, but the trial did not meet the predefined feasibility threshold of 95% of peak anti-Xa levels in the intervention group being less than the threshold for therapeutic anticoagulant effect (≤0.6 IU/mL). Of note, a weight-based dynamic algorithm was associated with fewer peak anti-Xa measurements below the prophylactic range (<0.2 IU/mL) when compared with standard care. The clinically observed proportions of deep venous thrombosis and pulmonary embolism were similar between groups, and bleeding events were infrequent, but this feasibility trial was not powered to detect differences in these clinical outcomes. Patient enrolment, randomisation and study drug prescription were embedded in the EMR, which was at least partially successful in aiding trial conduct.

A recent systematic review and meta-analysis including 39 studies (7124 patients) reported that both greater body weight and body mass index were associated with sub-prophylactic anti-Xa levels in critically ill adults receiving low molecular weight heparin thromboprophylaxis regimens.^33^ Furthermore, subprophylactic anti-Xa levels were associated with a nearly three-fold increased risk of venous thromboembolism. However, most studies used a prospective cohort design with only three randomised controlled trials included,^17^^,^^29^^,^^34^ none of which investigated a dynamic algorithm for venous thromboprophylaxis dosing based on anti-Xa levels. More than 50% of the included critically ill patients did not meet prophylactic steady-state anti-Xa targets despite adherence to common low molecular weight heparin thromboprophylaxis regimens.

There is limited evidence to inform the optimal dosing of low molecular weight heparin for venous thromboembolism prophylaxis in heavier critically ill patients. Previous studies of thromboprophylaxis in hospitalised or critically ill patients with obesity have evaluated fixed or weight-based regimens without dynamic adjustment based on anti-Xa levels and have not employed a randomised controlled trial design.[35], [36], [37], [38] Studies employing dynamic enoxaparin dosing guided by anti-Xa monitoring in critically ill patients have been predominantly single-centre and nonrandomised, with most recruited patients regardless of body weight. Such studies have enrolled bariatric surgery,^39^ trauma,[40], [41], [42] burns,^31^ colorectal surgery,^43^ and abdominal cancer surgery patients.^44^ These studies consistently identified a high proportion of patients with initial subprophylactic anti-Xa levels, which improved with dose adjustment. Dynamic dose adjustment based on anti-Xa level was associated with reduced rates of venous thromboembolism when compared with fixed-dose regimens in some,^40^^,^^42^^,^^44^ but not all,^41^ of these studies.

The trial has several limitations. Firstly, the randomisation process embedded into the EMR occurred after potential participants were screened as meeting all inclusion criteria. However, doctors had to review the exclusion criteria while unaware of treatment allocation. The final step included doctors becoming aware of treatment allocation and authorising the electronic prescription. This is an issue for many pragmatic trials embedded into the EMR,^27^^,^^28^ as both steps increase the risk of selection bias and unequal group numbers. To improve the internal validity of EMR-embedded trials, other researchers have customised randomisation engines into Epic.^45^ Since conducting this trial, we have linked the REDCap stratified randomisation module to Epic after exclusion criteria are checked for EMR embedded trials.^45^^,^^46^ While there were few obvious imbalances in baseline demographics for this trial, the risk of selection bias remains. Secondly, the trial utilised weight alone as an inclusion criterion, which may be an incomplete measure of obesity.^47^ Thirdly, we utilised peak anti-Xa ranges as previously defined in the literature,^30^^,^^31^ but acknowledge that expert opinion varies as to what the prophylactic peak anti-Xa range should be.^30^ We chose a single criterion rather than multiple criteria to assess efficacy and inform feasibility.^32^ Our rationale for using an anti-Xa level consistent with therapeutic anticoagulation as the threshold was that bleeding would be an infrequent event, yet if the risk of bleeding was perceived by clinicians as too great, clinicians would be unlikely to allow patient participation in a subsequent trial. It is important to emphasise that this criterion does not determine the safety or efficacy of our personalised approach to venous thromboprophylaxis. Furthermore, we also only measured peak, but not trough, anti-Xa levels, which limits the ability to build a comprehensive pharmacokinetic profile. Fourthly, despite embedding the trial in the EMR, some patients had missing anti-Xa levels due to incorrect timing of blood sampling by clinical staff. We described all missing data in detail. Fifth, the feasibility threshold for conducting a larger trial (95% of steady-state anti-Xa levels in the intervention group being ≤0.6 IU/mL, the threshold for therapeutic anticoagulant effect) was based only on the opinion of the trial team. It is possible that selecting a lower feasibility threshold, or utilising a more conservative dynamic enoxaparin dosing regimen in the intervention group, would make it feasible to conduct a larger trial. Sixth, clinical staff were not blinded to the intervention, and unblinded studies are at risk of outcome assessment bias. However, the primary outcome was the anti-Xa level, so the risk of ascertainment bias was low. Seventh, this trial excluded certain surgical patients at high risk of bleeding, and the trial data cannot be extrapolated to these groups.^48^ Similarly, by excluding patients with renal failure, the trial may have recruited a less ill cohort, and the trial findings may not be generalisable to patients with greater illness severity. Eighth, we assumed that three doses of enoxaparin would achieve a ‘steady state’ anti-Xa level, but this may not occur in all patients.^49^ Finally, this feasibility trial had a biochemical primary outcome and was not powered to evaluate patient-centred outcomes like thrombosis, bleeding, or mortality.

Given that one third of patients receiving standard care in this feasibility trial had anti-Xa levels below the prophylactic range at steady state, a larger future trial of a weight-based dynamic algorithm for venous thromboembolism prophylaxis in heavier critically ill patients appears warranted. Given that we did not meet our primary feasibility threshold of 95% of peak anti-Xa levels in the intervention group being less than the threshold for therapeutic anticoagulant effect (≤0.6 IU/mL), such a trial should investigate a different dosing algorithm than the one utilised in this feasibility trial. We recommend that future trials report incorrect dose administered, anti-Xa level not collected, or levels collected at an incorrect time as outcomes. Embedding such a trial in the EMR is likely to improve recruitment and protocol adherence. The EMR we used can prompt staff to comply with anti-Xa level monitoring and act on the result, but we conducted this trial relatively soon after Epic went live at our hospital. We speculate that as health systems gain greater familiarity with an EMR, and acceptance of embedded pragmatic trials, compliance will improve.^50^^,^^51^ Because a trial to assess patient-centred outcomes like thrombosis and bleeding is required to change practice, and this would require many ICUs and different EMRs, embedding the trial across sites is likely to be challenging. Planning for such a trial would require consideration of whether routine ultrasound is required to determine the true difference in the burden of thrombosis.^52^

## 5 Conclusions

A weight-based dynamic algorithm for pharmacological venous thromboembolism prophylaxis in heavier critically ill patients was embedded within the EMR and implemented within a randomised clinical trial, but this algorithm did not meet the predefined feasibility threshold.

## Authors’ contributions

YAA and AMD were responsible for study concept. YAA, AK, HP, TF, MA, LS, JA, CM, JP, and AMD were responsible for study design. YAA, FA, BT, TF and HP conducted data acquisition, analysis, and interpretation. HP and TF were responsible for design and configuration in the electronic medical record. NKP and AK conducted the statistical analyses. YAA and AMD drafted the manuscript. All authors substantively revised the manuscript, vouch for the accuracy and completeness of the data and fidelity of the trial protocol, and have approved the final submitted version of the manuscript.

## Consent for publication

Not applicable.

## Availability of data and materials

All data generated or analysed during this study are included in this published article and its supplementary information files.

## Funding

YAA is supported by a 10.13039/501100001782University of Melbourne Medical School Strategic Grant and 10.13039/501100000925National Health and Medical Research Council (10.13039/501100000925NHMRC) Investigator Grant (Emerging Leadership 1, ).GNT2041824

AMD is supported by a 10.13039/501100000925National Health and Medical Research Council (10.13039/501100000925NHMRC) Investigator Grant (Leadership 1) and the 10.13039/501100001782University of Melbourne Dame Kate 10.13039/100016655Campbell Fellowship (GNT2016819).

The trial was supported by an unrestricted grant from the Clive and Vera Ramaciotti Foundation to the senior author to support the embedding of pragmatic trials using electronic medical records into routine clinical care. The funder had no role in the design or conduct of the trial, collection of the data, or analysis, interpretation, and presentation of the results.

## Conflicts of interest

YAA and AMD declare that they are editorial board members for the journal ‘Critical Care and Resuscitation’. The other authors declare they have no competing interests.

## Footnotes

Supplementary data to this article can be found online at https://doi.org/10.1016/j.ccrj.2026.100229.
